# *Alphacoronavirus* in urban *Molossidae* and *Phyllostomidae* bats, Brazil

**DOI:** 10.1186/s12985-016-0569-4

**Published:** 2016-06-24

**Authors:** Karen Miyuki Asano, Aline Santana Hora, Karin Côrrea Scheffer, Willian Oliveira Fahl, Keila Iamamoto, Enio Mori, Paulo Eduardo Brandão

**Affiliations:** Instituto Pasteur, Av. Paulista, 393, CEP:01311-000 São Paulo, SP Brazil; Departament of Preventive Veterinary Medicine and Animal Health, School of Veterinary Medicine, University of São Paulo, Av. Orlando Marques de Paiva, 87, CEP: 05508-270 São Paulo, Brazil

**Keywords:** Bat, Coronavirus, *Molossidae*, *Phyllostomidae*

## Abstract

**Background:**

Bats have been implicated as the main reservoir of coronavirus (CoV). Thus the role of these hosts on the evolution and spread of CoVs currently deserve the attention of emerging diseases surveillance programs. On the view of the interest on and importance of CoVs in bats the occurrence and molecular characterization of CoV were conducted in bats from Brazil.

**Findings:**

Three hundred five enteric contents of 29 bat species were tested using a panCoV nested RT-PCR. Nine specimens were positive and eight was suitable for *RdRp* gene sequencing. *RdRp* gene phylogeny showed that all CoVs strains from this study cluster in *Alphacoronavirus* genus, with one *Molossidae* and one *Phlyllostomidae*-CoV specific groups. Phylogenetic analyses of two S gene sequences showed a large diversity within the *Alphacoronavirus* genus.

**Conclusions:**

This study indicated a CoV-to-host specificity and draws attention for CoV detection in *Cynomops sp*, a potential new reservoir. The phylogenetic analyses indicate that diversity of CoV in bats is higher than previously known.

## Background

Bats have been recognized as the natural reservoirs of a large variety of emerging and re-emerging viruses and have been implicated as the main reservoir of coronavirus (CoV). Thus the role of these hosts on the evolution and spread of CoVs currently deserve the attention of emerging diseases surveillance programs as illustrated by the finding of bats as reservoirs for SARS (Severe Acute Respiratory Syndrome) and MERS (Middle East Syndrome) coronavirus [1, 2].

CoV (*Nidovirales:Coronaviridae:Coronavirinae*) are classified into four genus: *Alpha* and *Betacoronavirus* are often found in mammals, while *Gammacoronavirus* were detected in wild birds, poultry, and marine mammals and *Deltacoronavirus* were detected in wild birds, pigs, and wild feline [3–5].

The first report of bat CoV was described in 2005 [6] in bats of *Miniopterus pusillus* species. Since then, several studies have identified the presence of CoV in bat population from various regions of the world, and to date have been detected both *Alphacoronavirus* and *Betacoronavirus* [6–13].

Although there is a great diversity of bats in Brazil, there are few studies related to bat CoV. *Betacoronavirus* has been reported in *a Desmodus rotundus* vampire bat and *Alphacoronaviruses* have been detected in *Molosuss rufus*, *M. currentium*, *M. molossus, Carollia perspicillata*, *C. brevicauda* and *Tadarida brasilensis* bats (14–18), but a range of bat species that might act as reservoirs for known or unknown CoVs still remains to be surveyed.

On the view of the interest on and importance of bat CoV surveillance, the aim of this study was survey the occurrence of bat CoV in Brazil and to perform molecular characterization of CoVs detected.

## Methods

This work was conducted with samples from 73 municipalities of São Paulo State, Southern Brazil, using 305 samples of enteric content of 29 bat species of three families (*Molossidae*, *Phyllostomidae* and *Vespertilionidae*). These animals were submitted to *Instituto Pasteur* (São Paulo, Brazil) from March/2013 to July/2014, as a part of rabies surveillance program, and were stored at −20 °C. Each animal was necropsied, the entire intestine was removed from abdominal cavity and all intestinal content was extracted and stored at −20 °C.

Feces suspensions (v/v, 10 %) were prepared with DEPC-treated water. Suspensions were clarified at 12,000 × *g* for 30 min at 4 °C and the supernatants were used in the assays. Extraction of total RNA was carried out with TRIzol Reagent™ (*Life Technologies*, Carlsbad, CA, USA) according to the manufacture’s instruction followed by reverse transcription with Random Primers and M-MLV™ *Reverse Transcriptase* (*Life Technologies*, Carlsbad, CA, USA*)* as per manufacturer’s instructions.

All samples were submitted to a pancoronavirus nested RT-PCR targeting the RNA-dependent RNA-polimerase (*RdRp*) [14], using *Taq Platinum™ DNA Polymerase* (*Life Technologies*, Carlsbad, CA, USA) as per manufacturer’s instructions. Positive samples were submitted to a RT-PCR targeting to S gene for phylogenetic inference [9].

Amplicons were purified using the ExoSap-IT® reagent (USB, Cleveland, OH, USA) or Illustra™ GFX™ Gel Extraction Kit (GE Healthcare, Buckinghamshire, UK) and bi-directional Sanger sequencing with the respective primers was carried out with BigDye v.3.1™ and ABI 3500 Genetic Analyzer™ (*Life Technologies*, Carlsbad, CA, USA).

Chromatograms generated were subjected to Phred online application (http://asparagin.cenargen.embrapa.br/phph/) for assessment of their quality and the final consensus sequences were obtained with CAP Contig application in Bioedit 7.2.5 program [15]. Sequences obtained were aligned with homologous sequences retrieved from GenBank using CLUSTAL/W software in Bioedit 7.2.5 program [15].

Alignments were used for phylogenetic trees construction with distance optimization criterion with neighbor-joining algorithm and Composite Maximum Likelihood evolutionary model with 1000 “bootstrap” repetitions, using the MEGA 6 program [16].

## Results and discussion

Nine out of the 305 samples (2,95 %) were found positive and DNA sequences were obtained for the *RdRp* for eight of them: sequences 4292/2013/*Desmodus rotundus*; 4539/2013/*Cynomops planirostris*, 4620/2013/*Glossophaga soricina*, 4702/2013/*Cynomops abrasus*, 4705/2013/*Cynomops abrasus*, 5026/2013/*Cynomops abrasus*, 2173/2014/*Cynomops planirostris*, 2218/2014/*Cynomops planirostris* (Genbank accession numbers KU552072 to KU552079).

Five species tested were positive: *Cynomops abrasus* (3/11 = 27.3 %)*, Cynomops planirostris* (3/5 = 60 %)*, Desmodus rotundus* (1/41 = 2.4 %)*, Glossophaga soricina* (1/33 = 3 %) and *Platyrrhinus lineatus* (1/12 = 8.3 %). Only the sample of *P. lineatus* was not confirmed by DNA sequencing.

The occurrence of CoV per specie demonstrates a high frequency in *Cynomops* genus: 60 % for *C. abrasus* and 27.3 % for *C. planirostris*, indicating these species must have an important role in CoV maintenance in bat population in this region. In South America, CoV detection on bats have shown a low occurrence of CoV per species [12, 17, 18], however, none of these studies included the *Cynomops* genus. Though CoVs have been described in Brazilian *Molossidae* bats [19, 20], *Cynomops sp* were previously unknown hosts for CoVs.

The *Alphacoronaviruses* found in *Cynomops sp* (*Molossidae*) were closely related with *Alphacoronaviruses* already detected in South Brazil (*RdRp* nt identities 81.4–82.9 %).

*D. rotundus* is one of the three hematophagous bat species and can be found only in the Americas, from northern Mexico to northern Argentina [21]. Although the presence of *Betacoronavirus* has already been described [22], this is the first report of an *Alphacoronavirus* in common vampire bat, showing that this species can carry both *Alpha* and *Betacoronavirus*.

*G. soricina* is a nectarivorous neotropical bat that might be found inside houses and has already been described as a host for *Alphacoronaviruses* [23]. Although separated by a large geographic area of more than 4000 km, *G. soricina* CoV found in São Paulo showed high nucleotide and amino acids identities when compared with *G. soricina* CoV of Trinidad and Tobago (90.3 and 98.4 %, respectively). *G. soricina* species does not migrate over long distances [24], therefore it is unlikely that transmission has occurred among these specimens, suggesting a virus-host adaptation.

**Fig. 1:**
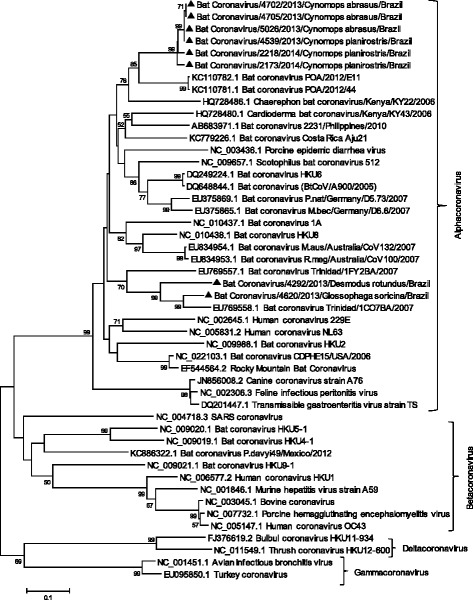
phylogenetic tree constructed with neighbor-joining method and maximum likelihood composite substitution model for partial 393 bp fragment of coronavirus *RdRp* gene. Numbers on each node represents the bootstrap values. The scale represents the number of substitutions sites. Samples of this study are identified with a black triangle

*RdRp* phylogeny (Fig. 1) showed that all CoVs strains from this study clustered in the *Alphacoronavirus* genus, with one *Molossidae* and one *Phyllostomidae*-CoV specific groups. The *Molossidae* CoV of this study clustered with *Molossidae* bat CoV from South Brazil with high bootstrap value (85 %). Although phylogenetic analysis indicates a geographic relation, the *Alphacoronaviruses* of this study was separated by a geographic area of 1000 km approximately from *Molossidae* batCoV previously detected in Brazil [19]. Besides, there are few studies regarding batCoV in Brazil, which difficult a phylogeographic relatedness hypothesis confirmation. *Phyllostomidae* CoV of this study clustered with sequences of *Carollia perspicillata* and *G. soricina*, two *Phyllostomid* bats from Trinidad and Tobago, with high bootstrap value (70 %) (Fig. 1). Taking together these results supports the theory that host specificity is more important than geographic pattern as previously noticed [13, 17, 25, 26].

Two 547 nt sequences of S gene (GenBank accession numbers KU552080 and KU552081) were obtained for *C. planirostris* and *C. abrasus* CoV. The nucleotide identity between these two sequences was high (99.8 %), suggesting the transmission of CoV among different bats species. However, the nucleotide identity with sequences retrieved from GenBank was low, varying from 39,1 and 65,8 %, showing a large genetic diversity with *Alphacoronavirus* from others countries.

**Fig. 2:**
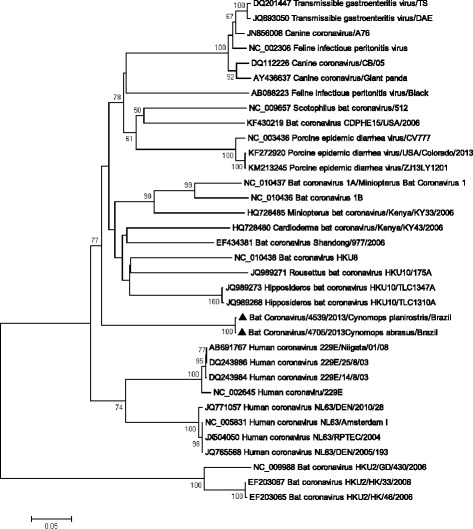
phylogenetic tree constructed with the neighbor-joining method and maximum likelihood composite substitution model for partial 547 bp fragment of coronavirus *S* gene. Numbers on each node represents the bootstrap values. The scale represents the number of substitutions sites. Samples of this study are identified with a black triangle

The phylogenetic tree performed for partial S gene (Fig. 2) shows that the samples of this study formed a separate group from others *Alphacoronavirus* sequences retrieved from GenBank, with bootstrap value of 100 %. Although the fragment analyzed was small, the tree indicates that samples of this study are unique, forming a completely separate group.

Environmental changes caused by man have promoted a major impact on ecology, affecting the movement of several wild animals species from their natural habitat to urban or rural areas [27], increasing the chances of contact between humans and domestic animals with wild animals. Furthermore approximately 75 % of emerging infectious diseases have zoonotic origin and wildlife as source of infection [28, 29]. Therefore, it is essential to survey and identify possible sources of infection, especially in relation to bats that are considered important reservoirs of viral agents [30, 31].

## Conclusion

The significance of CoVs detected during this survey on Public Health remains to be investigated, but the finding of CoV in new viral reservoirs justifies the need for CoV surveillance. This study indicates a CoV-to-host specificity and draws attention for CoV detection in *Cynomops sp*, suggesting the importance of this species for CoV maintenance in the region studied. The phylogenetic analyses indicate a great diversity of CoV in bats, particularly for S gene.

## Abbreviations

CoV: coronavirus; MERS: Middle East respiratory syndrome; RT-PCR: reverse transcription polymerase chain reaction; SARS: severe acute respiratory syndrome

## References

[1] Li W, Shi Z, Yu M, Ren W, Smith C, Epstein JH (2008). Bats are natural reservoirs of SARS-like coronaviruses. Science.

[2] Wang Q, Qi J, Yuan Y, Xuan Y, Han P, Wan Y (2014). Bat origins of MERS-CoV supported by bat coronavirus HKU4 usage of human receptor CD26. Cell Host Microbe.

[3] De Groot RJ, Baker SC, Baric R, Enjuanes L, Gorbalenya AE, Holmes KV, King A, Adams M, Cartens E, Lefkowitz E (2012). Family coronaviridae. Virus taxonomy: classification and nomenclature of viruses: ninth report of the International committee on taxonomy of viruses.

[4] Woo PCY, Lau SK, Lam CS, Lai KK, Huang Y, Lee P (2009). Comparative analysis of complete genome sequences of three avian coronaviruses reveals a novel group 3c coronavirus. J Virol.

[5] Woo PCY, Lau SK, Lam CS, Lau CC, Tsang AK, Lau JH (2012). Discovery of seven novel Mammalian and avian coronaviruses in the genus deltacoronavirus supports bat coronaviruses as the gene source of alphacoronavirus and betacoronavirus and avian coronaviruses as the gene source of gammacoronavirus and deltacoronavirus. J Virol.

[6] Poon LLM, Chu DK, Chan KH, Wong OK, Ellis TM, Leung YH (2005). Identification of a novel coronavirus in bats. J Virol.

[7] Tong S, Conrardy C, Ruone S, Kuzmin IV, Guo X, Tao Y (2009). Detection of novel SARS-like and other coronaviruses in bats from Kenya. Emerg Infect Dis.

[8] Osborne C, Cryan PM, O’Shea TJ, Oko LM, Ndaluka C, Calisher CH (2011). Alphacoronaviruses in new world bats: prevalence, persistence, phylogeny, and potential for interaction with humans. PLoS One.

[9] Shirato K, Maeda K, Tsuda S, Suzuki K, Watanabe S, Shimoda H (2012). Detection of bat coronaviruses from Miniopterus fuliginosus in Japan. Virus Genes.

[10] Dominguez SR (2007). O´Shea TJ, Oko LM, Holmes KV. Detection of Group 1 Coronaviruses in Bats in North America. Emerg Infect Dis.

[11] Lelli D, Papetti A, Sabelli C, Rosti E, Moreno A, Boniotti MB (2013). Detection of coronaviruses in bats of various species in Italy. Viruses.

[12] Moreira-Soto A, Taylor-Castillo L, Vargas-Vargas N, Rodríguez-Herrera B, Jimenez C, Corrales-Aguilar E (2015). Neotropical bats from Costa Rica harbour diverse coronaviruses. Zoonoses Public Health.

[13] Fischer K, Zeus V, Kwasnitschka L, Kerth G, Haase M, Groschup MH (2016). Insectivorous bats carry host specific astroviruses and coronaviruses across different regions in Germany. Infect Genet Evol.

[14] Chu DK, Leung CY, Gilbert M, Joyner PH, Ng EM, Tse TM (2011). Avian coronavirus in wild aquatic birds. J Virol.

[15] Hall TA (1999). BioEdit: a user-friendly biological sequence alignment editor and analysis program for Windows 95/98/NT. Nucleic Acids Symp Series.

[16] Tamura K, Stecher G, Peterson D, Filipski A, Kumar S (2013). MEGA6: molecular evolutionary genetics analysis version 6.0. Mol Biol Evol.

[17] Corman VM, Rasche A, Diallo TD, Cottontail VM, Stöcker A, Souza BF (2013). Highly diversified coronaviruses in neotropical bats. J Gen Virol.

[18] Góes LG, Ruvacaba SG, Campos AA, Queiroz LH, Carvalho C, Jerez JA (2013). Novel bat coronaviruses, Brazil and Mexico. Emerg Infect Dis.

[19] Lima FE, Campos DS, Kunert Filho HC, Batista HB, Carnielli P, Cibulski SP (2013). Detection of *Alphacoronavirus* in velvety free-tailed bats (*Molossus molossus*) and Brazilian free-tailed bats (*Tadarida brasiliensis*) from urban area of Southern Brazil. Virus Genes.

[20] Simas PV, Barnabé AC, Durães-Carvalho R, Neto DF, Caserta LC, Artacho L (2015). Bat coronavirus in Brazil related to appalachian rigde and porcine epidemic diarrhea viruses. Emerg Infect Dis.

[21] Greenhall AM, Joermann G, Schimidt U (1983). Desmodus rotundus. Mamm Species.

[22] Brandão PE, Scheffer K, Villarreal LY, Achkar S, Oliveira RN, Fahl WO (2008). A coronavirus detected in the vampire bat Desmodus rotundus. Braz J Infect Dis..

[23] Carrington CVF, Foster JE, Zhu HC, Zhang JX, Smith GJD, Thompson N (2008). Detection and phylogenetic analysis of group 1 coronaviruses in South American bats. Emerg Infect Dis.

[24] Fleming TH, Nuñez RA, Sternberg LSL (1993). Seasonal changes in the diets of migrant and non-migrant nectarivorous bats as revealed by carbon stable isotope analysis. Oecologia.

[25] Anthony SJ, Ojeda-Flores R, Rico-Chávez O, Navarrete-Macias I, Zambrana-Torrelio CM, Rostal MK (2013). Coronaviruses in bats from Mexico. J Gen Virol.

[26] Drexler JF, Gloza-Rausch F, Glende J, Corman VM, Muth D, Goettsche M (2010). Genomic characterization of severe acute respiratory syndrome-related coronavirus in European bats and classification of coronaviruses based on partial RNA-dependent RNA polymerase gene sequences. J Virol.

[27] Magle SB, Hunt VM, Vernon M, Crooks K (2012). Urban wildlife research: past, present, and future. Biol Conserv.

[28] Woolhouse ME (2002). Population biology of emerging and re-emerging pathogens. Trends Microbiol.

[29] Taylor LH, Latham SM, Woolhouse ME (2001). Risk factors for human disease emergence. Philos Trans R Soc Lond B Biol Sci.

[30] Wang LF, Walker PJ, Poon LL (2011). Mass extinctions, biodiversity and mitochondrial function: are bats ‘special’ as reservoirs for emerging viruses?. Curr Opin Virol.

[31] Wong S, Lau S, Woo P, Yuen KY (2007). Bats as a continuing source of emerging infections in humans. Rev Med Virol.

