# Erratum to: *Alphacoronavirus* in urban *Molossidae* and *Phyllostomidae* bats, Brazil

**DOI:** 10.1186/s12985-016-0581-8

**Published:** 2016-07-07

**Authors:** Karen Miyuki Asano, Aline Santana Hora, Karin Côrrea Scheffer, Willian Oliveira Fahl, Keila Iamamoto, Enio Mori, Paulo Eduardo Brandão

**Affiliations:** Instituto Pasteur, Av. Paulista, 393, São Paulo, SP CEP:01311-000 Brazil; Departament of Preventive Veterinary Medicine and Animal Health, School of Veterinary Medicine, University of São Paulo, Av. Orlando Marques de Paiva, 87, São Paulo, CEP: 05508-270 Brazil

## Erratum

Upon publication, it was noticed that the acknowledgements section of this article [1] should also include: The authors are grateful to Coordenacão de Aperfeiçoamento de Pessoal de Nível Superior (CAPES/PROEX 2327/2015) for financial support.
